# Comparison of Diagnostic Performance in Mammography Assessment: Radiologist with Reference to Clinical Information Versus Standalone Artificial Intelligence Detection

**DOI:** 10.3390/diagnostics13010117

**Published:** 2022-12-30

**Authors:** Won Jae Choi, Jin Kyung An, Jeong Joo Woo, Hee Yong Kwak

**Affiliations:** 1 Department of Radiology, Nowon Eulji University Hospital, Eulji University School of Medicine, Seoul 01830, Republic of Korea; 2 Department of Surgery, Nowon Eulji University Hospital, Eulji University School of Medicine, Seoul 01830, Republic of Korea

**Keywords:** artificial intelligence, breast neoplasm, mammography, radiologists

## Abstract

We compared diagnostic performances between radiologists with reference to clinical information and standalone artificial intelligence (AI) detection of breast cancer on digital mammography. This study included 392 women (average age: 57.3 ± 12.1 years, range: 30–94 years) diagnosed with malignancy between January 2010 and June 2021 who underwent digital mammography prior to biopsy. Two radiologists assessed mammographic findings based on clinical symptoms and prior mammography. All mammographies were analyzed via AI. Breast cancer detection performance was compared between radiologists and AI based on how the lesion location was concordant between each analysis method (radiologists or AI) and pathological results. Kappa coefficient was used to measure the concordance between radiologists or AI analysis and pathology results. Binominal logistic regression analysis was performed to identify factors influencing the concordance between radiologists’ analysis and pathology results. Overall, the concordance was higher in radiologists’ diagnosis than on AI analysis (kappa coefficient: 0.819 vs. 0.698). Impact of prior mammography (odds ratio (OR): 8.55, *p* < 0.001), clinical symptom (OR: 5.49, *p* < 0.001), and fatty breast density (OR: 5.18, *p* = 0.008) were important factors contributing to the concordance of lesion location between radiologists’ diagnosis and pathology results.

## 1. Introduction

Mammography, a basic imaging study for diagnosing breast cancer, has been used for a long time as a standard screening modality [1]. Although mammography increases cancer detection rate and reduces mortality rate, the possibility of missed diagnoses or false positives remains. Diagnostic performances of radiologists can also vary [2,3,4]. A computer-aided detection (CAD) technique has been introduced to facilitate mammography interpretation. CAD was approved by the U.S. Food and Drug Administration (FDA) in 1998 [5]. Since then, it has been widely used. Early studies have shown that traditional CAD might enable detection of microcalcifications and masses and result in reduced rate of false negatives [6]. However, its effectiveness in clinical setting is still controversial due to its low specificity, false-positive markings, and high recall rates [6,7,8,9].

Given the growing interest in the use of artificial intelligence (AI) in the medical field, several novel algorithms from around the world have been developed and trialed. Recent advances in convolutional neural networks (CNNs) and deep learning algorithms have led to a dramatic evolution and implementation of AI in the medical field [10,11]. A potential benefit of AI in the medical field has been suggested as radiological imaging data continue to grow disproportionate to the number of available trained readers. The use of AI can improve the sensitivity and specificity of lesion detection and shorten reading time [10,12,13,14,15].

Among many subspecialties in radiology, breast imaging is at the forefront of clinical applications of AI. The performance level of AI in mammography evaluation is comparable to that of experts [16,17,18,19,20,21]. It is expected that software for AI interpretation in conjunction with radiological evaluation can induce a double reading effect [22,23,24,25,26].

Lunit INSIGHT MMG (Lunit, Seoul, Korea), a software that aids breast cancer detection in a mammogram, was developed on the basis of deep convolutional neural net-works [16]. It used ResNet-34, one of the most popular CNN architectures, as a backbone network [27]. Lunit INSIGHT MMG used more than 200,000 cases analyzed in Korea, the United States, and the United Kingdom to train the AI algorithm. It has received authorization from the Korean Ministry of Food and Drug Safety.

Despite multiple benefits of AI, many papers report that additional tests, including prospective studies, are needed to apply AI to real clinical practice. For example, Yoon et al. reported that feasibility testing should be conducted while considering certain clinical aspects, such as incorporating of AI in clinical practice [11]. Wallis et al. reported that retrospective studies have failed to predict the real-world performance of radiologists and machines. Therefore, it is important to conduct a prospective study before introducing artificial intelligence into actual breast screening [28]. Sechopoulos et al. also emphasize large-scale screening trials to compare the performance of AI and breast screening radiologists in real-world screening domains [29].

In order to understand the limitation of AI and use it appropriately, it is important to consider differences between image analyses performed by AI and radiologists in real clinical environments. While a radiologist evaluates a mammography with reference to more clinical information, the AI reads only the image based on its own algorithm. Despite reports of increased cancer detection and decreased recall rate by AI, it is necessary to determine whether differences exist between the evaluation performed by AI and a radiologist in an actual clinical environment. Therefore, the objective of this study was to compared performances of radiologists and AI in breast cancer detection on digital mammography in real clinical practice.

## 2. Materials and Methods

### 2.1. Study Population

This retrospective study was reviewed and approved by our Institutional Review Board (IRB). The requirement for informed consent was waived by the IRB due to its retrospective nature. From January 2010 to June 2021, a total of 1314 patients underwent ultrasound-guided core biopsy in our hospital, of which 532 patients were diagnosed with malignancy. Among these patients, we excluded those without mammography (*n* = 52), those with film mammography (*n* = 14), and those with computed radiography (*n* = 74). Finally, this study included 392 women (average age: 57.3 ± 12.1 years, range: 30–94 years) diagnosed with malignancy who underwent digital mammography prior to biopsy. Each patient’s clinical symptoms (no symptom, palpation, pain, discharge, other), final pathological diagnostic method (biopsy, breast conservation surgery, or mastectomy), lesion location confirmed pathologically (right, left, both), and histology were reviewed based on their medical records.

### 2.2. Imaging Modalities

All mammographic examinations were bilateral and performed in four craniocaudal (CC) and mediolateral oblique (MLO) views. There were 378 mammographies performed in our hospital and 14 digital mammographies performed externally. Digital mammographic images in our hospital were obtained with a Selenia Full-Field Digital Mammography Unit (Hologic Inc.). Lunit INSIGHT MMG (version 1.1.3.0, Lunit), an artificial intelligence-based diagnostic software tool, was used to determine the probability of malignancy scores and markings of suspected lesion.

### 2.3. Imaging Analysis

Mammography was reviewed by two radiologists in consensus. Breast composition was evaluated according to the Breast Imaging Reporting and Data System (BI-RADS) (American College of Radiology). Breast compositions ‘a’ and ‘b’ were classified as fatty, while breast compositions ‘c’ and ‘d’ were classified as dense. Mammographic lesions were categorized into the following categories: invisible, mass, calcifications, mass with calcification, asymmetry, asymmetry with calcifications, architectural distortion, and others. The lesion location on mammography was classified as invisible, right, left, or both. In order to analyze the impact of previous mammography on the interpretation, the existence of comparable past mammography and its influence were observed. The effect of previous mammography was defined as follows: newly developed lesion, or interval change in size or density of previously noted lesion.

For AI analysis, 392 mammographies were interpreted using the Lunit system. In a study that validated vendor data, the probability of malignancy scores of 10% corresponded to AI’s breast cancer detection sensitivity of 90% and was used as the criterion for determining significance. Therefore, we adopted the score criterion, and a score below 10% was considered insignificant. AI displayed results of four standard mammographic views as values ranging between 0 and 100%. It visually highlighted each suspicious lesion with scores of 10% or higher. Based on the score criteria set at 10%, a lesion was diagnosed if any score in the CC or MLO view on each side was 10% or higher. However, if four views of both breasts were less than 10% without marking, it was classified as ‘invisible’. ‘Invisible’ means ’undetected’ in AI or radiologists’ analysis. This is a case where AI or a radiologist misses the lesion despite the presence of a lesion, or the lesion is not visible because it is masked by the breast parenchyma. The representative score of each case was determined using the largest score of the four views in each mammography.

For analysis, we used the probability of malignancy scores with the following quartile values, referring Lunit’s reader study [16,30]: below 10%, from 10% to less than 50%, from 50% to less than 90%, and 90% or higher.

### 2.4. Data Analysis

Clinicopathologic characteristics are expressed as mean and standard deviation for age or number with percentage for other features. Kappa coefficient was used to measure the concordance between radiologists’ diagnosis or AI analysis and pathology results. The strength of concordance was evaluated based on the following criteria: slight, kappa vale of 0–0.2; fair, kappa vale of 0.2–0.4; moderate, kappa vale of 0.4–0.6; substantial, kappa vale of 0.6–0.8; and almost perfect, kappa vale of 0.8–1.0 [31]. Binominal logistic regression analysis was performed to identify factors influencing the concordance of lesion location between radiologists’ analysis and pathology results. Statistical analyses were performed using Jamovi software (Version 1.2.22, Jamovi Project) [32]. Statistical significance was set at *p* < 0.05.

## 3. Results

### 3.1. Clinicopathological Characteristics

While the majority (212/392, 54.1%) of cases had no clinical symptoms, palpation of a mass was the most common symptom (153/392, 39%). A total of 242 patients underwent core biopsy alone and 150 underwent surgeries, including breast conserving surgery (90 cases) and mastectomy (60 cases). Similar proportions of lesions were located bilaterally (right, 202/392, 51.5%; left, 181/392, 46.2%). The most common histology was invasive ductal carcinoma (310/392, 79.1%), followed by ductal carcinoma in situ (42/392, 10.7%), invasive lobular carcinoma (12/392, 3.1%), mucinous or tubular carcinoma (8/392, 2% each), and other histologic types (12/392, 3.1%). Table 1 summarizes clinicopathological characteristics of patients.

### 3.2. Analysis of Mammography by Radiologists and AI

Breast composition ‘c’ was the most common breast density. Dense breast was found in a total of 282 (71.9%) cases. Old mammograms were present in 159 (40.6%) patients, with 103 (26.3%) cases influencing mammographic interpretation. The most common mammographic lesion type was ‘mass’ (142/392, 36.2%), followed by ‘calcifications’ (77/392, 19.6%). In terms of lesion location, there were 36 (9.2%) invisible cases in radiologists analysis compared with 57 (14.5%) cases in AI analysis. Majority (232/392, 59.2%) of cases showed lesion scores 90% or higher (Table 2).

### 3.3. Concordance of Lesion Location between Mammography and Pathology

The concordance of lesion location between mammography and pathology results was higher in radiologists’ analysis than in AI analysis of all cases (kappa = 0.819 vs. 0.698). In cases manifesting the effect of previous mammography, the concordance between radiologists’ analysis and pathological results was stronger than that between AI and pathology (kappa = 0.944 vs. 0.707). Similarly, radiologists’ analysis demonstrated an almost perfect concordance (kappa = 0.917) when patients had clinical symptoms. In terms of breast density, both radiologists’ diagnosis (kappa = 0.948 vs. 0.773) and AI analysis (kappa = 0.804 vs. 0.660) showed a higher level of concordance for fatty breast than for dense breast (Table 3).

### 3.4. Predictors of Concordance of Lesion Location with Pathology

In binomial logistic regression analysis, the effect of previous mammogram was a significant factor contributing to the concordance of lesion location with pathology (odds ratio (OR): 8.55; *p* < 0.001). Existence of symptoms also had a substantial effect on the concordance of lesion location with pathology (OR: 5.49; *p* < 0.001). When compared with dense breasts, fatty breasts were more consistent regarding lesion locations based on pathology (OR: 5.18; *p* = 0.008) (Table 4).

### 3.5. ‘Invisible’ Cases in Radiologists’ and AI Analyses

The number of cases with ‘invisible’ lesion type was 36 in radiologists’ analysis and 57 in the AI analysis. Four (11.1%) of these 36 invisible cases in radiologists’ analysis were concordant between AI analysis and pathology results, while 27 (47.4%) of 57 invisible cases in AI analysis were concordant between radiologists’ analysis and pathology results (Table 5).

Among these 27 concordant cases only in radiologists’ analysis, 13 cases showed the influence of previous mammography and 10 cases had clinical symptoms. The average size measured in mammography was 1.3 cm and the average AI score was 2.6% (Table 6).

**Figure 1:**
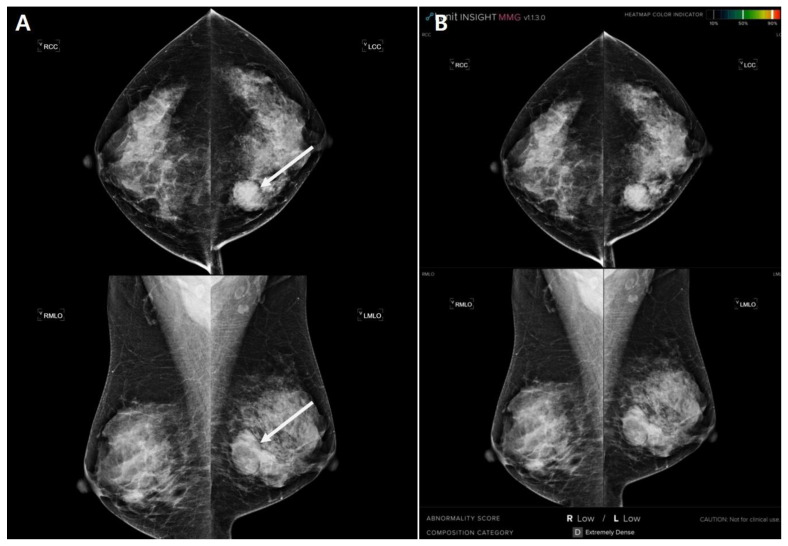
An invisible case in AI that is concordant only in radiologist’s analysis. A 53-year-old woman presented at the hospital with a palpable mass of her left breast. (**A**) Mammography showed a dense breast with a 3.7-cm-sized well-circumscribed oval mass in the left mediocentral breast (arrows). (**B**) Artificial intelligence revealed no abnormal lesion on the mammogram. The largest malignancy probability score was 0.85% of the left craniocaudal view and the abnormality score of the case was shown as ‘low’. The patient underwent ultrasound-guided core needle biopsy. She was diagnosed with invasive mucinous carcinoma.

**Figure 2:**
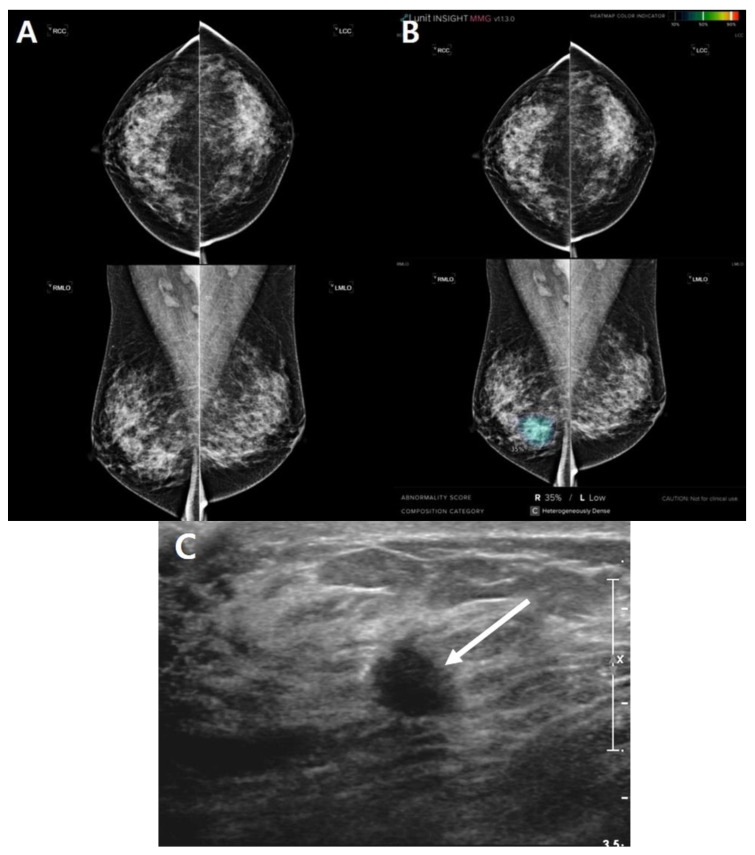
An invisible case in radiologist’s analysis that is concordant only in AI analysis. A 49-year-old woman had a breast checkup. (**A**) Mammography revealed a dense breast without any abnormal lesions in both breasts. (**B**) However, artificial intelligence highlighted a suspicious area on the right mediolateral oblique view and presents an abnormality score of 35%. (**C**) Ultrasound revealed a 0.8 cm irregular hypoechoic mass with angular margin in the right lower central breast (arrow). Ultrasound-guided core needle biopsy revealed invasive ductal carcinoma.

The largest lesion was a 3.7 cm oval mass and palpable (Figure 1). Four cases were concordant only in AI analysis. The mammography showed dense breasts and the average AI score was 26.3% (Table 7). The largest AI score was 35. 4% marked in a single mammographic view (Figure 2).

## 4. Discussion

Currently, many studies investigating the application of AI in mammography have reported decreases in false-positive and recall rate with an increase in cancer detection [16,22,24,33]. A reduction in radiologist’s workload is also expected [13,18,19,34]. However, image analysis via AI differs from the actual image reading performed by a radiologist. A radiologist’s analysis is not only based on images, but also based on additional information such as the patient’s clinical symptoms and comparison with previous mammography. A radiologist’s workload includes not only the number of mammographies to be read, but also the time required to evaluate the information in a single study. Therefore, we analyzed differences between radiologists’ analysis and AI analysis in real clinical practice and identified factors affecting the diagnostic performance of radiologists.

In our study, radiologists’ analysis was strongly consistent with pathology results in terms of overall lesion location. The number of cases with ‘invisible’ lesions was lower in radiologists’ analysis than in AI analysis. For ‘invisible’ cases, the concordance between radiologists’ analysis and pathology results was higher than that between AI analysis and pathology results.

The concordance strength was especially increased when the influence of old mammogram and clinical symptoms were present. First, the effect of old mammography was an important factor determining the concordance between radiologists’ analysis and pathological results. The concordance also increased compared with the case where no influence of an old mammography was detected. Thirteen of 27 cases that were concordant only in radiologists’ analysis were newly discovered or found due to an increase in size or density compared with previous mammography. In general, these lesions were small or showed subtle findings, suggesting that humans were more accurate than AI in evaluating and interpreting ambiguous mammographic findings based on old and recent studies. Rodriguez-Ruiz et al. [18] have also suggested that an ideal AI system should overcome limitations of the imaging method itself and detect occult cancer mammographically while minimizing false-positive results. Second, clinical symptoms were also important factors contributing to the concordance between radiologists’ analysis and pathological results. It is presumed that patients who experience clinical symptoms are more likely to manifest breast lesion progression, with a high probability of detection via imaging. Additionally, clinical information makes the radiologists carefully evaluate images, even when findings are minimal. Clinical symptoms were present in 10 of 27 cases that were concordant only in radiologists’ analysis. Among them, five cases showed small imaging findings with symptoms on palpation. The remaining 10 cases without symptoms or influence of old mammography showed small or subtle mammographic findings, which were undetected in AI analysis.

Both radiologists’ and AI analyses demonstrated poor concordance in lesions involving dense breasts than fatty breasts. Breast density is an independent risk factor for cancer by masking lesions in mammography [35,36,37]. Radiologists’ and AI analyses tended to reveal breast lesions better in fatty breasts than in dense breasts. Breast density was also an important contributing factor to the concordance of radiologists’ analysis with pathology results.

In our study, there was a lesion that evaded AI detection, although it was large and clearly visible. Lång et al. [38] have also reported a similar case. Figure 1 shows a case that could be identified by radiologists but missed by AI. The large mass in the left breast was apparent. However, the AI neglected the lesion and yielded a score of 0.85%, which could be explained by its somewhat benign looking oval and well-defined margin, which might have prompted the AI algorithm to perceive the lesion as benign. However, Figure 2 shows the ability of AI to detect tricky malignant findings that were missed by radiologists. Initially, radiologists assessed the mammogram as BI-RADS category 1, whereas the AI outlined the area of suspicion in the right breast and provided a lesion score of 35%. A subsequent ultrasound examination revealed an irregular hypoechoic mass of 0.8 cm in size in the right lower central breast, which was consistent with the location indicated by the AI. In a retrospective review, a subtle distortion in the breast parenchyma on the right medio-lateral oblique view was observed.

In our study, the detectability of AI was somewhat lower than that of radiologists. We used 10% as a valid set score of AI suggested by the manufacturer. Therefore, if the score was less than 10%, the marking was ‘invisible’ in AI analysis. However, a review of raw-score data revealed that in 22 of 27 cases detected only by radiologists, AI also gave a score less than 10% for the exact location of the lesion. With the low scores, the AI’s abnormal markings did not appear in the images. That is, if the 10% limit was not applied, AI could also detect the exact lesion location even in those 22 cases. This suggested that AI’s threshold score was not an absolute indicator of malignancy. In addition, an abnormality score ranging from 10 to 100 is rather broad for discriminating suspicious malignancies, underscoring the need for appropriate classification of scores generated by an AI algorithm.

Our study has several strengths and limitations. The strength was that we analyzed clinical symptoms and compared with a previous study to assess their influence on the accuracy of mammography interpretation by human experts. This allowed a more direct comparison between AI and radiologists analysis in real-world clinical setting. The limitation was that this study was performed retrospectively with a single AI vendor at a single medical center using a relatively small volume of data. Therefore, it is necessary to validate study findings with a larger population in the future.

In conclusion, radiologists’ interpretation of mammography in real clinical practice is superior to AI algorithm’s analysis in detecting breast cancer via digital mammography. Based on a comparison with previous study and reference to clinical symptoms, the evaluation by human experts significantly improved the accuracy of mammography interpretation. Although reducing the workload of radiologists through the triage of negative mammograms is one of the advantages of AI, AI has a limitation in that it cannot refer to various clinical information in the analysis process. Therefore, if the patient has symptoms or has had previous mammograms, confirmation by the radiologists should be considered, even if the AI classifies it as a negative mammogram. Breast density affected the detection of malignant lesions in both radiologists’ diagnosis and AI analyses. It is also necessary to validate the appropriate reference score for clinical use of AI.

## Figures and Tables

**Table 1:** Clinicopathological characteristics of study subjects.

Clinicopathological Characteristics	
Age (Years)	57.3 ± 12.1
Patient’s symptoms	
None	212 (54.1)
Palpation	153 (39.0)
Pain	13 (3.3)
Discharge	8 (2.0)
Others	6 (1.5)
Confirmation method	
Biopsy	242 (61.7)
BCS	90 (23.0)
Mastectomy	60 (15.3)
Location of pathologic lesion	
Right	202 (51.5)
Left	181 (46.2)
Both	9 (2.3)
Histology	
Invasive ductal carcinoma	310 (79.1)
Ductal carcinoma in situ	42 (10.7)
Invasive lobular carcinoma	12 (3.1)
Mucinous carcinoma	8 (2.0)
Tubular carcinoma	8 (2.0)
Invasive micropapillary carcinoma	3 (0.8)
Invasive tubulolobular carcinoma	2 (0.5)
Encapsulated papillary carcinoma	2 (0.5)
Metaplastic carcinoma	2 (0.5)
Adenoid cystic carcinoma	1 (0.3)
Papillary ductal carcinoma in situ	1 (0.3)
Lobular carcinoma in situ	1 (0.3)

Abbreviations: BCS, breast-conserving surgery. Data are presented as mean ± standard deviation for age or number (%) for categorical variables.

**Table 2:** Analysis of mammography by radiologists and AI.

Radiologists	
Breast density	
a	39 (9.9)
b	71 (18.1)
c	195 (49.7)
d	87 (22.2)
Presence of previous MG	
Nonexistent	233 (59.4)
Existent	159 (40.6)
Effects of past MG	
Nonexistence	289 (73.7)
Existence	103 (26.3)
Lesion type	
Invisible	36 (9.2)
Mass	142 (36.2)
Calcifications	77 (19.6)
Mass + Calcifications	61 (15.6)
Asymmetry	46 (11.7)
Distortion	20 (5.1)
Asymmetry + Calcifications	6 (1.5)
Other	4 (1.0)
Location of lesion	
Invisible	36 (9.2)
Right	178 (45.4)
Left	171 (43.6)
Both	7 (1.8)
**AI**	
Location of lesion	
Invisible	57 (14.5)
Right	163 (41.6)
Left	157 (40.1)
Both	15 (3.8)
Lesion score	
<10	57 (14.5)
10≤_<50	43 (11.0)
50≤_<90	60 (15.3)
90≤	232 (59.2)

Abbreviations: MG, mammography; AI, artificial intelligence.

**Table 3:** Concordance of lesion location between mammography and pathology.

		Analysis by	
	n	Radiologists	AI
		Kappa	
All	392	0.819	0.698
Surgical validation	150	0.833	0.701
Effect of previous MG			
Nonexistent	289	0.778	0.694
Existent	103	0.944	0.707
Symptoms			
Nonexistent	212	0.742	0.636
Existent	180	0.917	0.777
MG density			
Fatty (a, b)	110	0.948	0.804
Dense (c, d)	282	0.773	0.660

Abbreviations: MG, mammography; AI, artificial intelligence. Data include number of cases (n) and concordance with kappa coefficient.

**Table 4:** Binomial logistic regression analysis for predicting concordance of lesion location between radiologists’ analysis and pathology results.

Predictor	Estimate ^a^	Standard Error	*p*-Value	Odds Ratio	95% Confidence Interval	
					Lower	Upper
Previous MG influence (E/N)	2.146	0.625	<0.001	8.55	2.51	29.09
Symptoms (E/N)	1.703	0.422	<0.001	5.49	2.40	12.55
MG density (F/D)	1.644	0.622	0.008	5.18	1.53	17.51

Abbreviations: MG, mammography; E/N, ratio of existent to nonexistent reference; F/D, ratio of fatty breast to reference dense breast. ^a^ Estimates represent log odds of concordance vs. discordance.

**Table 5:** Concordance of ‘invisible’ lesions between pathology results and radiologists’ analysis or AI analysis.

Pathology	AI (Invisible in Radiologist)	Radiologists (Invisible in AI)
Concordance	4	27
Discordance	32	30
Total	36	57

ns: AI, Artificial intelligence. Data include number of cases.

**Table 6:** Invisible cases in AI and concordant cases only in radiologists’ analysis.

Case No.	Age (Year)	Symptom	Radiologists					AI	
			MG Density	Lesion Location	Lesion Type	Previous MG Influence	Lesion size on MG (cm)	AI Score (%)	Location by AI
R1	66	none	c	R	Asymmetry	-	0.5	8.46	R
R2	38	palpation	c	R	Calcification	-	0.4	0.1	L
R3	53	none	b	L	Asymmetry	Existence	0.4	1.3	L
R4	53	palpation	c	L	Mass	-	3.7	0.85	L
R5	67	pain	a	L	Asymmetry	-	1.6	2	L
R6	58	none	c	L	mass	Existence	0.7	7.5	L
R7	48	palpation	c	L	asymmetry	-	4	0.17	L
R8	40	palpation	d	R	asymmetry	-	3.2	0.28	R
R9	44	none	d	R	distortion	-	1.5	0.01	R
R10	45	none	d	R	calcification	-	1.6	6.53	R
R11	47	none	d	R	calcification	Existence	0.5	1.92	L
R12	45	palpation	d	R	mass	-	1.5	0.15	R
R13	51	none	c	R	calcification	-	0.5	3.5	R
R14	66	none	a	R	mass	Existence	0.6	0.01	L
R15	59	none	b	L	other	Existence	2.7	0.83	L
R16	66	discharge	c	L	asymmetry	Existence	0.7	3.5	L
R17	42	none	b	L	mass	-	1	0.68	L
R18	67	none	b	R	asymmetry	-	0.7	0.21	R
R19	46	palpation	c	L	mass	-	1.5	1.03	L
R20	56	none	c	L	mass	Existence	0.8	2.53	R
R21	70	none	c	L	distortion	Existence	1	5.27	L
R22	50	palpation	c	R	asymmetry	Existence	1	0.13	R
R23	47	none	d	L	calcification	-	0.5	6.29	L
R24	78	none	c	R	asymmetry	Existence	1	0.08	R
R25	46	none	c	L	distortion	Existence	0.8	8.72	L
R26	68	palpation	b	L	distortion	Existence	1.5	7.06	L
R27	59	none	c	R	asymmetry	Existence	1.4	0.34	L

Abbreviations: AI, Artificial intelligence; MG, mammography; L, left; R, right.

**Table 7:** Invisible cases in radiologist and concordant cases only in AI analysis.

Case No.	Age (Year)	Symptom	MG Density	AI Score (%)	Lesion Location
A1	35	Palpation	d	14.48	L
A2	49	Other	c	35.46	R
A3	46	Palpation	c	30.86	L
A4	42	Palpation	c	24.49	L

Abbreviations: AI, Artificial intelligence; MG, mammography; L, left; R, right.

## Data Availability

The data presented in this study are available upon reasonable request from the corresponding author.
